# Efficacy of metacognitive training on symptom severity, neurocognition and social cognition in patients with schizophrenia: A single‐blind randomized controlled trial

**DOI:** 10.1111/sjop.12811

**Published:** 2022-04-06

**Authors:** Zita Fekete, Edit Vass, Ramóna Balajthy, Ünige Tana, Attila Csaba Nagy, Barnabás Oláh, Nóra Domján, Ildikó Szabó Kuritárné

**Affiliations:** ^1^ Faculty of Medicine Institute of Behavioural Sciences, University of Debrecen Debrecen Hungary; ^2^ Doctoral School of Health Sciences University of Debrecen Debrecen Hungary; ^3^ Faculty of Medicine, Department of Psychiatry and Psychotherapy Semmelweis University Budapest Hungary; ^4^ Department of Psychiatry and Psychotherapy Jósa András Teaching Hospital, Szabolcs‐Szatmár‐Bereg County Hospitals and University Teaching Hospital Nyíregyháza Hungary; ^5^ Department of Psychiatric Rehabilitation URBS Pro Patiente Nonprofit Ltd. Budakalász Hungary; ^6^ Faculty of Public Health Faculty of Public Health Debrecen Hungary; ^7^ Faculty of Medicine, Department of Psychiatry University of Szeged Szeged Hungary

**Keywords:** Schizophrenia, metacognitive training, randomized controlled trial, neurocognition, social cognition

## Abstract

Over the past decades, a number of complementary treatments for schizophrenia have emerged. One of these is metacognitive training (MCT), which combines the principles of cognitive‐behavioral therapies, cognitive remediation, and psychoeducation into a hybrid approach placing emphasis on increasing metacognitive awareness. The aim of our study was to investigate the efficacy of MCT on symptom severity, and neurocognitive and social cognitive functioning in schizophrenia; also, attention was paid to the assessment of subjective acceptability. Forty‐six patients diagnosed with schizophrenia were included in our single‐blind randomized controlled trial, who were assigned to the intervention or control group. The intervention group was provided standard MCT, while the control group received treatment as usual. We assessed symptom severity and cognitive functions before and after the training, as well as after a 6‐month follow‐up period. Compared to the control group, the intervention group showed improvement in overall symptom severity, and positive and disorganized symptoms. Training participans showed further improvement at the follow‐up assessment. Regarding neurocognitive functions, improvement in visuospatial functions was observed between pre‐ and post‐intervention assessments compared to the control group. Patients showed excellent adherence, and evaluated the training as useful and interesting. In line with the results of previous studies, our results demonstrate the efficacy of MCT on symptom severity in schizophrenia. Improvements in cognitive functions that are closely related to the onset and prevalence of symptoms of schizophrenia were also found.

## INTRODUCTION

Increasing knowledge about cognitive impairment in schizophrenia has led to the emergence of a number of supplementary treatments in recent decades. Although antipsychotic treatment is the treatment of choice, professional guidelines currently support the use of psychologically based therapies (e.g., APA, 2020; NICE, 2014, 2020). Recommendations are primarily based on the cognitive behavioral therapeutic (CBT) approach, and also include cognitive remediation therapy (CRT). CBT aims to reframe the dysfunctional cognitive appraisals behind the symptoms of schizophrenia, and develop adaptive ways of behavior in addition to reducing maladaptive behavior (Morrison, 2009). On the other hand, CRT includes training programs aimed at improving neurocognitive processes affected in schizophrenia, by relying on scientific principles of learning (Bowie, Bell, Fiszdon *et al*., 2020; Wykes & Reeder, 2005).

Metacognitive training (MCT) was developed by Moritz and Woodward (2007); it is a hybrid method based on CBT that also employs CRT techniques and other techniques designed to improve social cognition. It also places strong emphasis on education. It is a small‐group, computer‐assisted training method that seeks to develop metacognitive awareness of cognitive impairments through targeted experiential tasks.

Metacognition, the function MCT is based on, can be defined as the knowledge of knowledge or cognition about cognitive phenomena (Flavell, 1979). Thus, metacognition is a function that acts as a monitor and controller over our cognitive processes (Nelson, 1996). According to the integrative model of metacognition developed by Lysaker (Lysaker & Hasson‐Ohayon, 2018), it can be interpreted as an umbrella concept with elements raging from discrete cognitive functions to complex and comprehensive cognitive functions which also includes neurocognitive and social cognitive functions (Fleming, Dolan & Frith, 2012; Lysaker & Dimaggio, 2014).

There is evidence for the fact that better metacognitive functioning is associated with better neurocognitive performance in schizophrenia – these results were summarized in a metaanalysis by Davies and Greenwood (2018), and recently confirmed by a 12‐month follow‐up study by Kukla and Lysaker (2020). Similarly, there is evidence that performance on social cognitive tasks and metacognitive performance are actually related (Kukla & Lysaker, 2020; Lysaker, Vohs, Ballard *et al*., 2013). Neurocognitive impairments are core features of schizophrenia, where mostly attention, memory, executive functions, language, processing speed, and spatial functions are adversely affected (Kalkstein, Hurford & Gur, 2010; Kéri, Kiss, Kelemen, Benedek & Janka, 2005).

In addition to neurocognitive deficits in schizophrenia, a large body of evidence has been found in recent decades to account for deficits in social cognition. Its subdomains are emotion recognition, theory of mind, attributional style, and social perception; and patients with schizophrenia show impairments in all of them (Green, Horan & Lee, 2015; Pinkham, Penn, Green, Buck, Healey & Harvey, 2014). The contribution of social cognitive deficits to the emergence and maintenance of schizophrenia symptoms is well established (e.g., Abu‐Akel, 1999; Brüne, 2005; Frith, 2004; Hardy‐Baylé, Sarfati & Passerieux, 2003; Kelemen, 2019). This body of evidence led to the development of supplementary treatments in this field (Vass, Fekete, Simon & Simon, 2018), where we can find treatments specifically targeting one of the subdomains of social cognition (e.g., Theory of Mind Intervention [ToMI], Bechi, Spangaro, Bosia *et al*., 2013; Training of Affect Recognition [TAR], Wölwer & Frommann, 2011), or others that comprehensively target impairments in social cognition in schizophrenia (e.g., Social Cognition and Interaction Training [SCIT], Roberts & Penn, 2009).

Impairments in metacognition have been determined to contribute to the development and persistance of schizophrenia symptoms (Lysaker, Kukla, Dubreucq *et al*., 2015). In this regard, we have data not only on the relationship between metacognition and general symptom severity, but also on the association between the impaired metacognitive functioning and the severity of the symptomatic categories of schizophrenia, such as positive symptoms (Arnon‐Ribenfeld, Hasson‐Ohayon, Lavidor, Atzil‐Slonim & Lysaker, 2017), negative symptoms (Lysaker *et al*., 2015; McLeod, Gumley & Schwannauer, 2014), or symptoms of disorganization (Hamm, Renard & Fogley, 2012).

Metacognitive training aims to improve metacognitive function by helping patients to increase awareness of their cognitive processes, which is expected to improve symptom severity, especially the positive symptoms of schizophrenia (Moritz & Woodward, 2007).

Several studies have shown the efficacy of MCT on symptom severity in schizophrenia; these effects have been demonstrated in several trials controlled not only with treatment‐as‐usual, but also with active control (for meta‐analyses see e.g., Eichner & Berna, 2016; Liu, Tang, Hung, Tsai & Lin, 2018). Most of these studies focused on the positive symptoms of schizophrenia, but there are also favorable results on the effect of MCT on the general symptom severity of schizophrenia (Aghotor, Pfueller, Moritz, Weisbrod & Roesch‐Ely, 2010; Pankowski, Kowalski & Gaweda, 2016). In addition, Naughton, Nulty, Abidin, Davoren, O'Dwyer and Kennedy (2012) found marginally significant improvement regarding negative symptoms.

The primary aim of the present study was to investigate the effect of MCT on symptom severity, whereas the secondary objective was to study the effect of MCT on neurocognitive and social cognitive functions as well. Our third objective was to test the feasibility and subjective applicability of the training in a population of patients diagnosed with schizophrenia.

To this end, we designed a single‐blind randomized controlled trial with a 6‐month follow‐up period. The results were reported in line with the CONSORT 2010 (Consolidated Standards of Reporting Trials) statement (Schulz, Altman & Moher, 2011).

## METHODS

### Participants

We used convenience sampling to recruit participants: patients with schizophrenia diagnosis were referred into the study by their psychiatrist. Psychiatrists only referred patients into the study who fulfilled the DSM‐5 diagnostic criteria for schizophrenia based on the available illness history and clinical exploratory data. Subsequently, we relied on the clinical diagnoses set up by psychiatrists. Patients could naturally voluntarily decide whether or not they wished to participate in the study.

Inclusion criteria included diagnosis of schizophrenia (DSM‐5), general intelligence above 70 IQ points determined by the Wechsler's Adult Intelligence Scale (WAIS) (Kun & Szegedi, 1983; Wechsler, 1944), and in line with the recommendations of the applied training method, the absence of explicit antisocial, indiscrete, sexual, or hostile behavior.

**Fig. 1:**
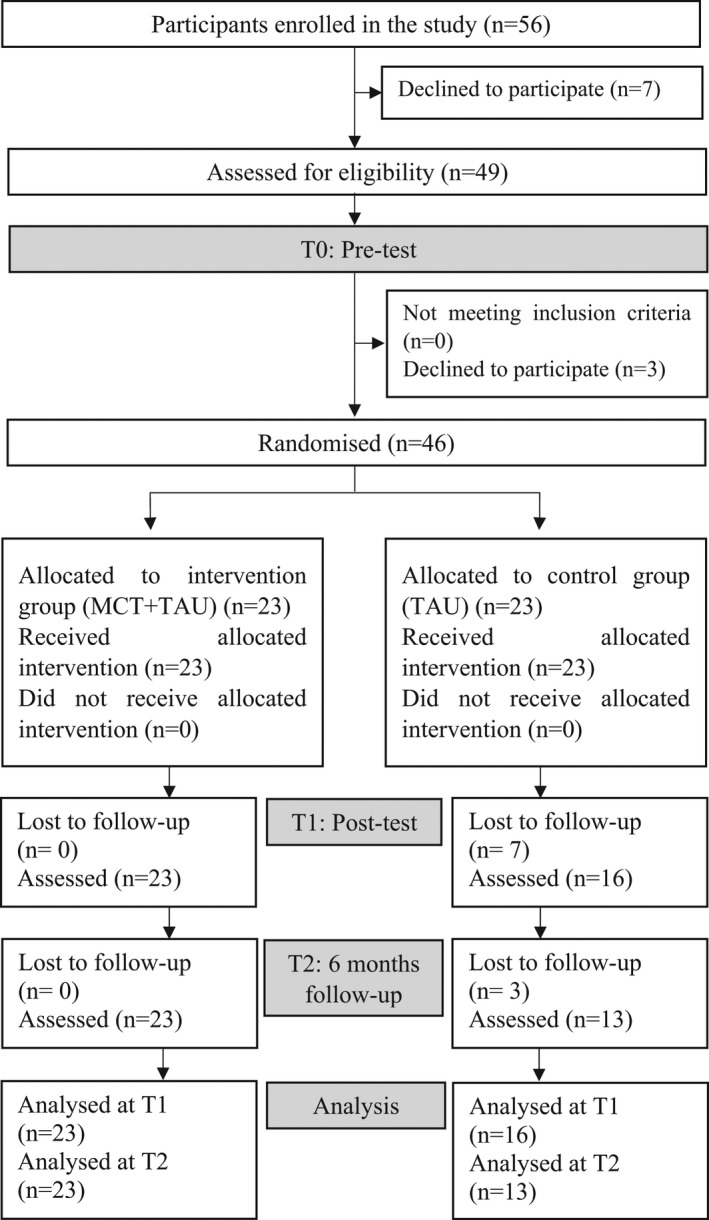
Flow diagram of the study progress in line with the CONSORT 2010 statement. MCT, metacognitive training; TAU, treatment as usual.

Fifty‐six patients accepted their psychiatrist's recommendations and agreed to participate in the study at three study sites, but 10 of them withdrew their consent before or during the pre‐test measurement process. Thus, the data of these 10 patients were withdrawn from the study. Accordingly, we started the present study with 46 patients. All of them met the inclusion criteria for IQ and behavioral characteristics. The participants were in remission according to the criteria of Lehman and van Os: they were on stable antipsychotic medication, and no medication change was needed in the previous 6 months (Lehman, Lieberman, Dixon *et al*., 2004). Most of them showed delusions, unusual thought content, hallucinatory behavior, disorganization, mannerism, blunted affect, passive/apathetic social withdrawal, and lack of spontaneity only to a moderate extent based on the Positive and Negative Syndrome Scale for Schizophrenia (PANSS) scores (<3) (van Os, Burns, Cavallaro *et al*., 2006) at enrollment. Two of the patients showed disorganized symptoms; delusional fragments were explored in 10 patients with PANSS scores below 3 points regarding the items of delusions, unusual thought content, and disorganization. According to their psychiatrists, these symptoms did not require hospitalization or changes in medication. Seven patients in the control group left the study prematurely, at the time of the post‐test assessment, and three further patients decided not to take part in the follow‐up assessment. None of the participants in the intervention group discontinued the study (Fig. 1).

#### Ethics

The Hungarian National Research Ethics Committee approved the study protocol under authorization number 13175‐2/2017/EKU. All patients signed an informed consent form, and were assured of anonymity and data confidentiality. They participated voluntarily in the study. Patients were randomly allocated either into the intervention group or the control group. The participants in the control group also had the opportunity to take part in the training after the follow‐up phase of the study, if they decided so.

### Design and procedures

To study the efficacy of MCT (Moritz & Woodward, 2007) on symptom severity, and neurocognitive and social cognitive functions in our sample of patients diagnosed with schizophrenia, we designed a single‐blind randomized controlled study. Three study sites enrolled participants into the study; however, all of the participants at one of the sites left the study prematurely. This site only enrolled four patients, who later declined to participate, either before or during the screening phase. Subsequently, we conducted the study at two study sites, and had 36 participants at one site, and 10 participants at the other.

Participants were randomly assigned into the treatment or the control group. Participants in the treatment group took part in a standard MCT along with psychiatric treatment as usual (TAU, psychopharmacological therapy and regular psychiatric control and care), while patients in the control group only received TAU. Standard MCT lasted for 16 weeks (one session per week). Participants took part in the training in small groups (four to five patients in each group). All participants received all the modules of standard MCT. Assessment of symptom severity, neurocognition and social cognition was conducted before (T0) and after the training (T1), and after a 6‐month follow‐up period (T2) by a blinded clinical psychologist at each site. The blinded assessors had not checked patients' documentation before the assessment, received patients allocated for assessment by the study coordinator; and the training sessions took place at locations other than the examining psychologists' offices to avoid the assessors meeting training participants. The study sites had their own trainers and assessors do the job; trainers were different from the assessors. Both trainers and assessors had been thoroughly trained on the applied training method and assessment tools before the start of the study. Measurements were carried out by postgraduate trained and licensed clinical psychologists at each study site. The trainer at one of the sites was a licensed clinical psychologist and psychotherapist, while at the other site there was a psychiatrist acting as trainer. All professionals in the study were trained by the first author, a licensed clinical psychologist and psychotherapist to ensure the uniformity of the assessments and MCT training processes across the study sites.

Altogether, 10 months elapsed between T0 and T2 assessment. The study was conducted between 2016 and 2020.

The same assessment was carried out at all the three time points (symptom severity, neurocognitive and social cognitive functions).

#### Randomization

To ensure the blinding of the study and patients' anonymity, all participants got an identification number based on the order of their inclusion. Randomization was done at the pace of inclusion, with 10–15 individuals per step. To avoid biases that might arise from the unequal distribution of patients' symptom severity, stratified randomization was used after the pre‐test. Randomization was conducted by the study coordinators at the study sites. Stratification was done along the PANSS score of 75 (Leucht *et al*., 2005). For the detailed description of the randomization process see the Supplementary Material A.

### Measures

#### General intelligence

We used WAIS (Kun & Szegedi, 1983; Wechsler, 1944) to measure participants' intelligence when screening them for inclusion criteria. WAIS is a widely used tool for the assessment of general intelligence, and consists of two parts with five subtests each. One part is for measuring verbal intelligence with the subtests named Comprehension, Arithmetics, Information, Digit‐span, and Similarities, and another part for performance IQ with the subtests entitled Picture Arrangement, Picture Completion, Blocks, Object Assembly, and Digit Symbol. All the 10 subtests were administered to the patients, using the Hungarian version of WAIS (Kun & Szegedi, 1983).

#### Symptom severity

The *PANSS* (Kay, Fiszbein & Opler, 1987; Kay, 1991) was used in our study for assessing symptom severity. It includes a structured interview, the data of which are evaluated by the administering clinician on a 30‐item scale with individual item scores from one to seven. For scoring we adopted the scoring method of van der Gaag, Hoffman, Remijsen *et al*. (2006). It is a five‐factor model of PANSS, which proved to be more stable, and gives a more nuanced picture of the symptoms and symptom severity in schizophrenia than the widely used three‐factor model. The five factors are the following: positive symptoms (with the maximum achievable score of 49 points), negative symptoms (max. 56 points), disorganization (max. 70 points), excitement (max. 56 points), and emotional distress (max. 56 points). Van der Gaag *et al*. recommend the classification of certain items into more than one subscales. This is because certain phenomena described in the items may not only be present within a single symptomatic category (e.g. uncooperativeness may be present in states of excitement, but may also be an inherent part of negative symptoms). However, they do not give a recommendation for the calculation of the total score. We computed total PANSS values according to the traditional scoring method (Kay *et al*., 1987) by the summation of the scores of the items. The highest possible score was 210 points, which indicates the worst possible symptom severity.

#### Neurocognition

To assess executive functions, the shortened version of the Wisconsin Card Sorting Test was applied using 64 cards (*WCST‐64*) (Axelrod, Woodard & Henry, 1992; Heaton, 1981). The test assesses mental flexibility. The task of the subject is to group cards with different figures, colors, and number of figures, according to a given sorting principle that is unknown for the subject, and should be found out on the basis of the feedback provided by the assessor.

WCST is a commonly used assessment tool for executive functioning in schizophrenia research (e.g., Carruthers, Gurvich, Meyer *et al*., 2019; Kurtz, Moberg, Gur & Gur, 2001; Nieuwenstein, Aleman & de Haan, 2001). We can gain information regarding total errors, perseverative responses, perseverative errors, conceptual level responses, and the number of categories completed. In this study we focused on the number of errors and perseverative errors, since the results of the other three outcome data depend on the amount of errors.

To assess some further neuropsychological functions of our patients we employed the Repeatable Battery for the Assessment of Neuropsychological Status (RBANS) (Randolph, Tierney, Mohr & Chase, 1998) test. The battery is designed for neuropsychological screening and identifying cognitive deficits. It provides scores for achievement related to five subcales, namely Immediate Memory (measured with the subtests List Learning and Story Immediate Memory); Visuospatial Functions (Figure Copy and Line Orientation); Language (Picture Naming and Semantic Fluency); Attention (Digit Span and Coding); and Delayed Memory (List Recall, List Recognition, Story Delayed Recall and Figure Recall). Each subscale represents a specific cognitive domain. The maximum of raw scores for the best performance in each subscale is 64, 60, 50, 105, and 82, respectively, with a total of 361 points. The test has an age standard, but only the raw scores for the subscales were used in the current study.

The utility of RBANS as an effective assessment tool in schizophrenia has been confirmed in many studies (e.g., Dickerson, Boronow, Stallings, Origoni, Cole & Yolken, 2004; Loughland, Lewin, Carr, Sheedy & Harris, 2007; Wilk, 2004).

#### Social cognition

Emotion recognition was assessed with the help of the Reading the Mind in the Eyes Test (RMET) (Baron‐Cohen, Wheelwright, Hill, Raste & Plumb, 2001). In this test, 36 pictures representing eyes of female and male faces are presented, which show different emotions with four response options per trial. The task of the subject is to identify the emotion. One point is given for each correct answer. A maximum of 36 points can be collected.

Theory of Mind Picture Stories Task (ToM PST) (Brüne, 2003) evaluates the ability to infer mental states to others, and predict their behavior on different levels of intentionality. First, a short cartoon story is presented in four pictures in the wrong order. The task is to sequence the pictures correctly. Then the rater asks pre‐defined questions regarding first‐order, second‐order, and third‐order beliefs, and false beliefs in connection with the stories. For the scoring of the answers we used our own scoring method, which showed appropriate psychometric properties on a sample of schizophrenic patients (Fekete, Vass, Balajthy *et al*., 2021a). It contains the following four scales: Sequencing, Theory of a Single Person's Mind, Switching Between Minds, and Comprehension of Misleading. For the best possible performance subjects can achieve 59 points overall.

#### Hospital admissions

A further outcome measure of our study was the number of days spent in hospital due to the illness. Data on hospital admissions were retrieved from the medical documentation of the participants at the end of the 6‐months follow‐up period.

#### Subjective acceptance and feasibility

The efficacy of the training to be evaluated by group members was measured by asking ten questions relevant from the aspect of psychological interventions (e.g. usefulness in daily life, whether they would recommend it to others, etc.) The questions of Moritz, Kerstan, Veckenstedt *et al*. (2011) were used with the author's permission. The items were rated on a five‐point Likert scale.

### Intervention method

The intervention method employed in our study was MCT for psychosis, which was developed by Moritz and Woodward (2007). MCT aims to improve the metacognitive and social cognitive functions of patients, and subsequently reduce symptom severity. It is a computer‐assisted group training program. In the first part of the training sessions, relevant psychotic symptoms related cognitive phenomena are highlighted with the help of psycho‐educational elements displayed on Power Point slides; then targeted tasks are displayed. These are playful tasks using photos, paintings, short cartoon stories, or examples from everyday life to make patients aware of common cognitive failures, and train their metacognitive and social cognitive skills. Some of the tasks have pre‐defined options in the form of multiple choice questions, while others do not, but all of the tasks can only be solved after mobilizing metacognitive functions. Group sessions are complemented with exercises to be done on worksheets at home. Standard MCT contains two cycles with eight modules (attributional style, jumping to conclusions, belief inflexibility, theory of mind, overconfidence in memory errors, depressed mood) in each. In addition, MCT contains two extra modules (self‐esteem and stigma). These modules can be used in an embedded form in other modules. The extra modules were not applied in our present research because they were not yet available in Hungarian at the start of the study.

### Data analysis

Normality was evaluated with the help of the Kolmogorov–Smirnov test. Comparisons between dichotomous variables were conducted using the chi‐squared test or Fisher's exact test. The Mann–Whitney *U‐*test or independent samples *t*‐test was used for independent samples, based on the normal or non‐normal distribution of data. To study the relationship between a dependent variable and one or more explanatory variables linear regression analyses were computed.

As we have measurements obtained at up to three time points for each patient, multilevel analysis was conducted. Originally, measurements of 19 variables were planned to be conducted on each participant at all the three time points (T0: baseline, T1: post‐test, T2: follow‐up). However, drop‐outs led to a large amount of missing data (30.44% at T1; 43.48% at T2). Subsequently, data imputation was not carried out, but a robust method was used for statistical analysis instead. All available measurements were included in the analyses. Data were analysed using generalized linear mixed models due to the ability of the model to handle the lacking data and different types of variables simultaneously at multiple levels (McGilchrist, 1994). Models analyzing changes within treatment conditions over time included time (T0, T1, and T2) as fixed factor with fixed intercepts, and subjects as a random factor. To select the best‐fitting model, fixed and random intercept models were compared. Differences in changes between treatment conditions included treatment condition, the time of assessment, and the interaction between treatment condition and time, as predictors, as well as a relevant covariant (duration of illness – the only value that proved to be marginally significant when comparing the intervention group and control group at baseline). Subject numbers served as a random factor. This complex model was set up in several steps. First, models with interaction terms were compared to models without interaction terms. Second, models with random intercepts and fixed intercepts were compared.

Model fit was evaluated in all cases using Akaike's information criterion (AIC), which is based on log likehood and the simplicity of the model. Therefore, it is a commonly used tool for model selection and evaluation of goodness‐of‐fit (Cavanaugh & Neath, 2019).

To adjust for type I error rate, we corrected our results using Bonferroni's correction method for multiple comparisons. The procedure applies the following formula for adjust significance level: *p* < *α*/*m* where *m* denotes the number of investigated endpoints (Goeman & Solari, 2014).

Data were analysed using SPSS version 23.0 (IBM Corp, Armonk, NY) for Windows. The database used was Fekete, Vass, Balajthy *et al*. (2021b).

## RESULTS

Considering the whole sample, nearly half of the study participants were male (*n* = 22, 47.83%), and the mean age of the participants was 41.30 years (*SD* = 10.73, range: 18–60 years). They had been suffering from schizophrenia symptoms for 13.74 years on average (*SD* = 8.53, range: 1–34 years), and had been hospitalized due to the illness 6.28 times on average (*SD* = 4.52, range: 1–33 times). More than two thirds had at least a high school certificate (*n* = 37, 80.43%), and almost all were single at the start of the study (*n* = 44, 95.65%); two thirds were employed (*n* = 32, 74.42%). The mean PANSS total score of the sample was 80.24 (*SD* = 22.56), and the mean IQ was 105.65 (*SD* = 13.37). The mean olanzapine equivalent dose according to the method of defined daily doses was 12.83 mg/day (*SD* = 7.84) (Leucht, Samara, Heres & Davis, 2016). When comparing baseline demographic data at randomization we found that participants in the TAU and TAU + MCT groups did not differ regarding their demographic data, PANSS symptom severity scores, general intelligence, or olanzapine equivalent dose. However, the statistical comparison of the duration of the illness remained only marginally above the pre‐defined level of significance (Table 1).

**Table 1:** Baseline demographic and clinical data in the total sample, and separately for the intervention and control group

	Total *n* = 46	TAU *n* = 23	MCT + TAU *n* = 23	*t*/*U*	χ^2^ (*df*)	*p*
Male, *n* (%)	22 (47.83)	11 (47.83)	11 (47.83)	‐	<0.00 (1)	1.000^a^
Age, mean (*SD*, Mdn)	41.30 (10.73, 40)	38.39 (10.41, 39)	44.22 (10.45, 4)	1.89	‐	0.065^b^
Duration of illness, mean (*SD*, Mdn)	13.74 (8.53, 14.5)	11.32 (8.74, 9.5)	16.16 (7.76, 16)	1.94	‐	0.059^b^
Number of hospital admissions, mean (*SD*, Mdn)	7.25 (6.24, 5)	9.0 (8.20, 5)	5.82 (3.61, 5)	165.0	‐	0.367^c^
Educational attainment				‐	‐	1.000^d^
Elementary or vocational school, *n* (%)	9 (19.57)	5 (21.74)	4 (17.39)			
High school or university, *n* (%)	37 (80.43)	18 (78.26)	19 (82.61)			
Marital status				‐	‐	0.489^d^
Single or divorced, *n* (%)	44 (95.65)	23 (100)	21 (91.30)			
Married or in a relationship, *n* (%)	2 (4.35)	0 (0)	2 (8.70)			
Occupational status				‐	0.411(1)	0.522^a^
Unemployed, *n* (%)	14 (30.43)	8 (34.78)	6 (26.09)			
Employed, *n* (%)	32 (69.57)	15 (65.22)	17 (73.91)			
PANSS total, mean (*SD*, Mdn)	80.24 (22.56, 78.5)	80.87 (20.43, 77)	79.61 (24.96, 80)	0.19	‐	0.852^b^
IQ, mean (*SD*, Mdn)	105.65 (13.37, 106)	103.22 (12.54, 102)	108.09 (14.00, 110)	1.24	‐	0.221^b^
OLA (mg/day), mean (*SD*, Mdn)	12.83 (7.84, 12.58)	11.43 (6.20, 10)	14.26 (9.40, 13.33)	1.19	‐	0.242^b^

*Notes*: IQ = intelligence quotient; MCT = metacognitive training; Mdn = median; OLA = olanzapine equivalents according to the DDD (defined daily doses) method (Leucht *et al*., 2016). PANSS = Positive and Negative Syndrome Scale; SD = standard deviation; TAU = treatment as usual.

^a^ Chi‐squared test.

^b^ *t*‐test.

^c^ Mann–Whitney *U* test.

^d^ Fischer's exact test.

Participants in the intervention group attended 97.55% of the group settings (total number of settings multipled by the total presence of TAU + MCT group members resulted in 359 attendances and nine absences). All the TAU + MCT group participants completed the study (100%), while seven participants in the TAU group left the study by T1 (drop‐out rate: 15.22% for the whole sample), and three further participants left by T2 (drop‐out rate: 21.74% for the whole sample). As data reveal, treatment condition definitely affected the drop‐out rate; and to investigate whether other factors may have also had a role, we calculated multiple linear regressions. Here we used the baseline demographic variables, treatment condition, and symptom severity as covariates. We found that apart from treatment condition the duration of illness also predicted study continuation, as a significant regression equation was found there (*F*[1, 45] = 9.574, *p* < 0.001) with an *R* ^2^ of 0.318. This means that more chronically ill patients in the control group tended to discontinue the study before the final assessment.

When investigating within‐group changes, evaluations of goodness of fit showed best model fit in the case of models with fixed intercepts. The best model fit occured in the case of models for between‐groups changes using interaction terms for all items except for the subscales of delayed memory (RBANS) and theory of a single person's mind (ToM PST). In addition, the fixed intercept model was found to be appropriate in all cases except for perseverative errors (WCST) and sequencing (ToM PST). Detailed results on AICs are displayed in Supplementary Material B.

Results were corrected for multiple comparisons using Bonferroni correction. Three out of the 19 investigated variables are the composite of the values of other variables (PANSS total, WCST total errors, and ToM PST total), so they cannot be considered as independent measures. Accordingly, we calculated the Bonferroni correction equation with 16 endpoints (*m* = 16), and the procedure gave an adjusted significance level of *α* = 0.0031.

### Symptom severity

Our primary outcome was change in symptom severity. Comparisons between T0 and T1 are presented in Table 2, while comparisons between T1 and T2 can be found in Table 3. When comparing intervention group and control group (*B* = −14.34, *p* = 0.026), an improvement in overall symptom severity was found in favor of the TAU + MCT group (*B* = −14.34, *p* = 0.026). A further improvement could again be detected during the 6 months follow‐up period (*B* = −14.95, *p* = 0.033). Within‐group changes further confirm these results (TAU + MCT from T0 to T1: *B* = −10.44, *p* = 0.029).

**Table 2:** Within‐group and between‐group differences between pre‐test (T0) and post‐test (T1) measurements

	TAU		TAU + MCT		Statistics							
					within group				Between groups			
					TAU/TAU + MCT							
	T0 mean (*SD*) *n* = 23	T1 mean (*SD*) *n* = 16	T0 mean (*SD*) *n* = 23	T1 mean (*SD*) *n* = 23	*B*	CI	*F*	*p*	*B*	CI	*F*	*p*
*Symptom severity* PANSS												
Positive	20.30 (7.24)	21.13 (6.53)	18.61 (7.60)	16.57 (7.20)	0.20/−2.04	−2.62 to 3.01/−5.21 to 1.12	0.02/1.69	0.884/0.200	−4.66	−9.21 to −0.10	3.29	**0.045**
Negative	21.13 (7.45)	20.44 (6.74)	22.96 (9.80)	19.09 (7.40)	−0.33/−3.84	−3.83 to 3.12/−7.70 to − 0.43	0.04/1.15	0.851/**0.048**	−1.92	−2.88 to 6.73	0.10	0.428
Disorganized	26.30 (9.22)	27.63 (8.03)	26.57 (8.22)	22.0 (6.80)	0.67/−4.57	−1.95 to 3.30/−7.83 to −1.30	0.27/7.94	0.607/**0.007**	−5.98	−10.92 to −1.04	1.79	**0.018**
Excitement	19.04 (5.37)	19.38 (4.23)	17.52 (5.87)	16.22 (5.45)	0.119/−1.30	−1.97 to 2.21/−4.26 to 1.65	0.01/0.79	0.909/0.379	−3.10	−6.54 to 0.35	3.01	0.077
Emotional distress	23.22 (6.26)	22.56 (6.22)	22.70 (8.70)	20.09 (6.40)	−0.73/−2.61	−3.32 to 1.86/−5.91 to 0.70	0.33/2.53	0.572/0.119	−3.27	−7.45 to 0.90	1.92	0.122
Total	80.87 (20.43)	81.88 (16.41)	79.61 (24.96)	69.17 (20.44)	0.41/−10.44	−5.57 to 6.40/−19.77 to −1.10	0.02/5.07	0.889/**0.029**	−14.34	−26.88 to −1.80	2.33	**0.026**
*Neurocognition* Executive functions (WCST‐64)												
Total error	23.57 (12.31)	28.25 (11.81)	25.13 (10.66)	24.74 (14.15)	4.77/−0.24	−1.29 to 10.83/−6.24 to 5.76	2.55/.006	.119/.937	−3.35	−12.38 to 5.69	0.18	0.463
Perseverative error	7.17 (7.36)	5.5 (6.25)	7.96 (6.47)	5.22 (5.98)	−2.06/−3.24	−4.76 to .66/−7.21 to .73	2.36/2.71	.133/.107	−.82	−4.31 to 2.68	0.12	0.643
Further neurocognitive functions (RBANS)												
Immediate memory	40.78 (8.61)	43.25 (8.80)	43.43 (8.19)	43.87 (7.81)	2.20/0.44	−1.61 to 6.01/−1.81 to 2.68	1.37/.15	.250/.698	1.14	−4.27 to 6.55	0.51	0.675
Visuospatial functions	34.83 (5.13)	35.25 (4.97)	35.13 (5.67)	37.52 (2.95)	.52/2.34	−1.03 to 2.07/0.24 to 4.54	0.47/5.03	0.498/**0.030**	2.71	0.30 to 5.11	1.95	**0.028**
Language	28.65 (5.52)	32.0 (5.83)	26.48 (5.40)	28.26 (5.90)	3.16/1.78	0.94 to 5.31/−0.78 to 4.35	8.42/1.96	**0.006**/0.168	−3.82	−7.69 to 0.05	4.25	0.053
Attention	56.70 (19.05)	53.87 (18.02)	47.87 (13.03)	50.39 (11.60)	−4.52/2.52	−9.95 to 0.91/−0.57 to 5.61	2.85/2.71	0.100/0.107	−3.45	−12.08 to 5.18	2.58	0.428
Delayed memory	43.87 (10.70)	48.25 (9.84)	45.96 (7.87)	48.78 (7.56)	3.96/2.83	1.48 to 6.44/0.93 to 4.73	10.47/8.99	**0.003**/**0.004**	0.784	−4.40 to 5.96	0.09	0.764
*Social cognition* Emotion recognition (RMET)												
Emotion recognition	21.83 (4.23)	21.87 (3.87)	20.91 (4.99)	20.61 (4.62)	0.29/−0.30	−2.27 to 2.33/−2.12 to 1.50	0.001/0.12	0.980/0.735	−0.96	−3.94 to 2.02	0.89	0.348
Theory of mind (ToM PST)												
Sequencing	26.0 (7.83	27.17 (5.79)	29.43 (7.62)	30.87 (5.58)	1.47/1.06	−0.87 to 3.81/−0.71 to 3.37	1.63/3.23	0.210/0.076	3.61	−0.34 to 7.55	3.32	0.072
Theory of a single person's mind	9.04 (2.51)	9.47 (2.97)	10.04 (2.69)	10.78 (2.21)	0.40/0.74	−0.53 to 1.33/−0.30 to 1.78	0.76/2.06	0.389/0.152	1.39	−0.63 to 3.40	2.31	0.174
Switching between minds	1.57 (1.24)	1.33 (1.23)	1.39 (1.16)	2.09 (1.16)	−0.40/0.70	−1.27 to 0.59/0.21 to 1.17	0.55/8.48	0.463/**0.006**	0.71	−0.11 to 1.54	0.75	0.090
Comprehension of misleading	5.7 (1.26	5.93 (1.58)	5.78 (1.62)	6.43 (1.31)	0.23/0.65	−0.74 to 1.19/0.15 to 1.15	0.22/6.89	0.641/**0.012**	0.61	−0.38 to 1.60	0.64	0.226
Total	42.3 (11.09)	44.13 (9.88)	46.65 (11.46)	50.26 (10.17)	2.0/3.61	−0.60 to 4.60/0.03 to 7.19	2.43/4.13	0.128/**0.048**	6.60	0.13 to 13.32	3.16	0.054

*Notes*: Bold: significant before Bonferroni correction (*α* = 0.05).

CI = confidence interval; MCT = Metacognitive Training; PANSS = Positive and Negative Symptoms Scale; RBANS = Repeatable Battery for the Assessment of Neuropsychological Status; RMET = Reading the Mind in the Eyes Test; SD = standard deviation; TAU = treatment as usual; ToM PST = Theory of Mind Picture Stories Task; WCST = Wisconsin Card Sorting Test.

**Table 3:** Within‐group and between‐group differences between post‐test (T1) and follow‐up (T2) measurements

	TAU		TAU + MCT		Statistics							
					within group				Between groups			
					TAU/TAU + MCT							
	T1 mean (SD) *n* = 16	T2 mean (SD) *n* = 13	T1 mean (SD) *n* = 23	T2 mean (SD) *n* = 23	*B*	CI	*F*	*p*	*B*	CI	*F*	*p*
*Symptom severity* PANSS												
Positive	21.13 (6.53)	18.77 (8.14)	16.57 (7.20)	15.00 (5.78)	−1.67/−1.57	−3.73 to 0.40/−4.0 to 0.86	2.73/1.69	0.110/0.200	−4.78	−9.48 to −0.08	4.12	**0.046**
Negative	20.44 (6.74)	19.92 (8.67)	19.09 (7.40)	18.65 (8.15)	−0.59/−0.44	−4.80 to 3.63/−3.03 to 2.16	0.08/0.11	0.778/0.737	−1.83	−7.67 to 4.02	0.39	0.535
Disorganized	27.63 (8.03)	27.69 (10.00)	22.0 (6.80)	21.83 (7.07)	−0.02/−0.17	−6.70 to 6.62/−2.95 to 2.60	2.23/0.02	0.996/0.900	−6.89	−12.75 to −1.02	5.49	**0.022**
Excitement	19.38 (4.23)	18.00 (5.18)	16.22 (5.45)	14.91 (3.93)	−0.96/−1.30	−2.94 to 1.02/−3.18 to 0.57	0.10/1.97	0.327/0.167	−3.15	−6.36 to 0.05	3.85	0.054
Emotional distress	22.56 (6.22)	21.23 (8.21)	20.09 (6.40)	18.26 (6.45)	−1.58/−1.83	−4.92 to 1.76/−4.10 to 0.44	0.95/2.63	0.339/0.112	−3.43	−8.40 to 1.54	1.90	0.173
Total	81.88 (16.41)	77.08 (23.28)	69.17 (20.44)	64.52 (16.94)	−3.81/−4.65	−13.31 to 5.71/−10.60 to 1.30	0.67/2.48	0.419/0.122	−14.95	−28.67 to −1.23	4.73	**0.033**
*Neurocognition* Executive functions (WCST‐64)												
Total error	28.25 (11.81)	23.62 (12.72)	24.74 (14.15)	24.48 (13.29)	−4.91/−0.26	−10.02 to 0.20/−3.76 to 3.24	3.90/0.023	0.059/0.881	1.23	−7.37 to 9.82	0.08	0.777
Perseverative error	5.5 (6.25)	5.85 (4.69)	5.22 (5.98)	5.13 (5.91)	.036/−0.09	−2.25 to 2.98/−2.94 to 2.77	0.08/0.004	0.778/0.951	−0.57	−4.29 to 3.16	0.09	0.762
Further neurocognitive functions (RBANS)												
Immediate memory	43.25 (8.80)	44.14 (10.21)	43.87 (7.81)	47.70 (9.19)	2.26/3.83	−1.68 to 6.19/1.41 to 6.24	1.38/10.19	0.250/**0.003**	1.31	−4.94 to 7.57	0.18	0.676
Visuospatial functions	35.25 (4.97)	35.36 (4.83)	37.52 (2.95)	37.48 (2.98)	0.34/−0.43	−1.42 to 2.10/−1.07 to 0.98	0.47/0.007	0.693/0.932	2.24	−0.21 to 4.69	3.33	0.073
Language	32.0 (5.83)	32.0 (7.17)	28.26 (5.90)	30.57 (7.10)	1.14/2.30	−1.69 to 3.97/−0.45 to 4.16	0.68/6.24	0.415/**0.016**	−2.82	−7.44 to 1.79	1.49	0.226
Attention	53.87 (18.02)	55.86 (19.49)	50.39 (11.60)	49.52 (12.11)	2.34/−0.87	−0.87 to 5.56/−3.95 to 2.21	2.23/0.33	0.146/0.572	−7.27	−17.33 to 2.79	2.08	0.154
Delayed memory	48.25 (9.84)	48.21 (8.98)	48.78 (7.56)	50.96 (8.40)	1.13/2.17	−1.15 to 3.40/0.03 to 4.32	1.03/4.17	0.318/**0.047**	1.14	−4.37 to 6.66	0.17	0.680
*Social cognition* Emotion recognition (RMET)												
Emotion recognition	21.87 (3.87)	19.92 (5.88)	20.61 (4.62)	20.96 (5.28)	−2.46/0.35	−4.99 to 0.06/−1.16 to 1.85	4.02/0.22	0.056/0.643	1.06	−2.84 to 4.96	0.30	0.589
Theory of mind (ToM PST)												
Sequencing	27.17 (5.79)	27.36 (9.00)	30.87 (5.58)	32.09 (6.71)	0.50/1.22	−3.89 to 2.89/−0.80 to 3.24	0.09/1.48	0.764/0.231	−3.61	−18.40 to 11.18	0.24	0.628
Theory of a single person's mind	9.47 (2.97)	10.14 (2.35)	10.78 (2.21)	10.96 (2.92)	0.61/0.15	−0.34 to 1.57/‐ 0.47 to 0.82	1.72/0.30	0.201/0.588	0.88	‐ 0.10 to 2.75	0.87	0.353
Switching between minds	1.33 (1.23)	1.86 (1.23)	2.09 (1.16)	2.09 (1.16)	0.59/−4.44	−0.04 to 1.22/−0.34 to 0.34	3.69/7.29	0.065/1.000	0.02	−0.80 to 0.84	0.003	0.958
Comprehension of misleading	5.93 (1.58)	5.79 (1.58)	6.43 (1.31)	6.13 (1.77)	−0.85/−0.30	−0.59 to 0.42/−0.69 to 0.09	0.12/2.48	0.729/0.122	0.15	−1.01 to 1.31	0.07	0.798
Total	44.13 (9.88)	45.21 (5.88)	50.26 (10.17)	51.30 (11.62)	0.64/1.04	−3.79 to 5.08/−1.56 to 3.64	0.09/0.66	0.768/0.423	5.34	−2.65 to 13.32	1.78	0.187

*Notes*: Bold: significant before Bonferroni correction (*α* = 0.05).

CI = confidence interval; MCT = Metacognitive Training; PANSS = Positive and Negative Symptoms Scale; RBANS = Repeatable Battery for the Assessment of Neuropsychological Status; RMET = Reading the Mind in the Eyes Test; SD = standard deviation; TAU = treatment as usual; ToM PST = Theory of Mind Picture Stories Task; WCST = Wisconsin Card Sorting Test.

Linear regression was calculated to predict the extent of change in overall symptom severity (PANSS total scores) based on the baseline PANSS scores (≥75 PANSS total score or <75 PANSS total score) of the patients in the TAU + MCT group. Results showed that patients with a baseline PANSS total score ≥75 presented a greater improvement in symptom severity compared to patients with a lower symptom severity at baseline (T0–T1: (*F*(1, 21) = 15.550, *p* <0.001, *R* ^2^ = 0.425, *B* = −21.800; and (T0–T2: (*F*(1, 21) = 4.501, *p* = 0.046, *R* ^2^ = 0.177, *B* = −16.938).

When considering the subscales of PANSS a decline in positive symptoms was seen between T0 and T1 in favor of the TAU + MCT group (*B* = −4.66, *p* = 0.045), and a further difference between the groups was detectable between the end‐of‐training and the follow‐up assessments (*B* = −4.78, *p* = 0.046). The scores of disorganized symptoms of the PANSS also improved in the TAU + MCT group between T0 and T1 (*B* = −5.98, *p* = 0.018) compared to the control group. This difference was still detectable 6 months after the training (*B* = −6.89, *p* = 0.018).

### Neurocognition

Cognitive outcomes were assessed as secondary outcomes. No relevant differences between the TAU and the TAU + MCT group were detected at any assessment point, either in terms of total errors or perseverative errors (WCST).

The TAU + MCT group showed an improvement compared to the TAU group in visuospatial functions between T0 and T1 (*B* = 2.71, *p* = 0.028); this effect, however, did not persist into the follow‐up assessment (*B* = 2.41, *p* = 0.073).

Looking at the results within the group, we managed to detect improvements in language (*B* = 3.16, *p* = 0.006) and delayed memory (*B* = 2.83, *p* = 0.004) in the TAU + MCT group, and improvement in delayed memory (*B* = 3.96, *p* = 0.003) in the TAU group between T0 and T1. The results regarding delayed memory proved to be significant in the TAU group and marginally significant in the TAU + MCT group at the adjusted significance level. A further improvement of these functions could be detected in the TAU + MCT group between T1 and T2 (language: *B* = 2.30, *p* = 0,016; delayed memory: *B* = 2.17, *p* = 0.047). Furthermore, the improvement in immediate memory proved to be significant in the TAU + MCT group during the follow‐up period (*B* = 3.83, *p* = 0.003).

### Social cognition

No significant change in emotion recognition (RMET) was found either within or between groups at the post‐test and the follow‐up assessment.

The TAU + MCT group showed improvements in the “Switching Between Minds” (*B* = 0.70, *p* = 0.006) and “Comprehension of Misleading” (*B* = 0.65, *p* = 0.012) scales of ToM PST, and produced a better overall theory of mind performance (*B* = 3.61, *p* = 0.048) between T0 and T1. These effects were not detectable at T2, though, or between the TAU and TAU + MCT groups.

It is important to note that the majority of our results remain above the adjusted α.

### Hospital admissions

Participants in the TAU + MCT group had an average of 3.5 (*SD* = 6.01) days of hospital admission during the 10 months of the trial, while participants in the TAU group had an average of 12.39 (*SD* = 13.67) days of hospital admission, which proved to be statistically significant at a large effect size (*U* = 219.00, *p* = 0.048, Cohen's *d* = 0.842).

Also, we found evidence for the fact that MCT has beneficial effects on real‐life adaptation. These results are published elsewhere (Fekete, Vass, Balajthy *et al*., 2021c).

### Subjective acceptance and feasibility

More than half of the participants of MCT totally agreed that the training was useful (*n* = 12, 52.17%); it was an important part of their treatment (*n* = 12, 52.17%), and it was fun (*n* = 12, 52.17%). Nearly two thirds totally agreed they would recommend the training to others (*n* = 16, 69.57%), and also found it advantageous that the training was administered in a group setting (*n* = 16, 69.57%). Nearly half of the participants found the sessions totally useful for their daily routine (*n* = 11, 47.83%), and considered the goals of the training crystal clear (*n* = 11, 47.83%). Half of the subjects did not have to force themselves to go to the training (*n* = 12, 52.17%), and would not have preferred to spend the time doing something else (*n* = 14, 60.87%). Only two (8.7%) of the 23 participants totally agreed that they would not apply the lessons learnt in their everyday lives.

### Adverse events

On the eighth week of the training, one patient (female, aged 38) requested admission to hospital due to depressive symptoms. The patient related this event to what she had experienced during the training. She said, “I knew I had a problem, but I have just become aware of how bad I really am at relating to people.” She was hospitalized for 10 days with depressive symptoms. This made her miss two sessions, but when she returned to the group, she again took part intensively in the training, showing considerable motivation.

## DISCUSSION

Reviewing our results we could conclude that there were notable improvements in various domains in favor of the TAU + MCT group; some of the results remained significant even after using Bonferroni correction for multiple comparisons with a strict threshold of significance. It is well known that the higher the number of tested variables, the bigger the chance of committing a type I error. At the same time, this also serves as the root of common criticisms of the correction procedures, since the interpretation of the results along the mentioned procedures depends heavily on the number of tests performed (Perneger, 1998). In addition, setting a threshold below which a type I error is not committed, does not exclude the chances of committing a type II error. It cannot be stated that all results above the adjusted threshold are false positives (VanderWeele & Mathur, 2019). Thus, completely ignoring results that fall between the significance level of 0.05 and the adjusted threshold may result in losing important data. Therefore, we recommend a more comprehensive review of our study results on symptom severity and cognitive functions, even if they should be handled with caution.

### Symptom severity

Looking at the results for overall symptom severity, we can see that compared to the TAU group, the TAU + MCT group showed a considerable improvement by T1, and this effect was still evident 6 months after the end of the training (T2). Moreover, our results indicate that patients in the TAU + MCT group with more severe symptoms benefitted more from the training regarding the severity of their symptoms. These results could be seen not only at the end of the training, but 6 months later as well.

The TAU + MCT group also showed a notable improvement in positive symptoms at T1 compared to the TAU group. Six months after the training, this difference was still detectable between the groups. In addition, the TAU + MCT group was superior to the TAU group in improved disorganized symptoms; this difference was detected both at T1 and T2.

It is also important to note that within the TAU + MCT group improvement in negative symptoms was found between T0 and T1, while this was not true within the TAU group.

Results on overall symptom severity and the positive and disorganized symptoms may imply a long‐lasting effect of training on schizophrenia symptomatology. Even if *p* values remain above the adjusted significance level, our results show a trend consistent with the findings of previous studies (e.g., Eichner & Berna, 2016; Liu *et al*., 2018).

### Neurocognition

In terms of executive functions, we could not find any relevant differences between the intervention and control group, or within the groups. However, even if our results are not statistically relevant, it is worth looking at the WCTS‐64 results. We can see that at T0 the TAU + MCT group produced slightly more total errors. The reason behind it might be the following: the fact that we failed to find any relevant difference in the extent of changes between the groups at assessments T1 and T2 does not only mean there was no change in the quality of executive functions, but also reveals that intervention group members “caught up.” This is quite important, as ToM processing in schizophrenia is related to executive function deficits (Hardy‐Bayléas cited at Brüne, 2005), and ToM deficits contribute to the onset and prevalence of symptoms (e.g., Abu‐Akel, 1999; Frith, 2004; Kelemen, 2019). In addition, deficits in executive function may be related to symptoms of disorganization in schizophrenia (Hardy‐Baylé *et al*., 2003). Subsequently, improvements in executive function may be expected to be associated with symptom remission.

Group members performed significantly better during the follow‐up period compared to their own performance at earlier assessments. The modules for metamemory of MCT aim to reduce metamemory biases (e.g., false memories or response confidence) common in schizophrenia (Moritz & Woodward, 2007). For this aim the training contains tasks that require immediate recall Accordingly, MCT may contribute to the improvement of immediate memory. Neverteless, this difference was not found significant when comparing the TAU + MCT and TAU groups.

In terms of other memory functions, our results are contradictory. We found improvements regarding delayed memory both in the TAU and TAU + MCT groups, but no significant differences were seen between the two groups. This is regarded to be rooted in a learning effect phenomenon. RBANS contains many subtests that can be completed in a short period of time, so subjects may have remembered some of the tasks from the previous assessment(s).

Finally, the TAU + MCT group showed an improvement in visuospatial function between T0 and T1 compared to the TAU group. Visuospatial inattention is related to impaired illness awareness (Curtin, Sun, Zhao *et al*., 2019; Daniell, Kim, Iwata *et al*., 2021). For this reason, our results may potentially imply improved awareness, which has been associated with improvements in metacognition (Lysaker, Dimaggio, Buck *et al*., 2011). This cannot be verified, though, as we did not aim to measure awareness. Furthermore, there are data on the correlation between perceptual organization deficits, and negative and hallucinatory symptoms (Kéri *et al*., 2005).

### Social cognition

No significant difference was found in emotion recognition between the two groups. RMET scores did not significantly differ between the TAU and TAU + MCT groups, either between T0 and T1, or between T0 and T2. The reason behind this may be that although MCT employs some tasks aimed at emotion recognition, the more explicit focus is on ToM functions. Accordingly, we found an improvement regarding ToM within the TAU + MCT group. Members of the TAU + MCT group showed a considerable improvement on the “Switching Between Minds” scale of ToM PST, that is, the scale measuring third‐order ToM processes (T0–T1). Similarly, better performance was found on the “Comprehension of Misleading” scale, which measures higher‐order theory of mind functions as well. The TAU + MCT group showed an improvement in overall ToM (T0–T1), while no improvement was detectable within the TAU group. However, it is important to note that these differences were not detectable when comparing the two groups at any time points, and the mentioned results did not show up in the post‐training period. At the same time, our results can be of importance, as the trait‐like ToM impairment in schizophrenia is well known, it is present even in the remissive stages of the illness (Herold, Tényi, Lénárd & Trixler, 2002; Kelemen, 2019). Alterations in ToM functions play a marked role in the prevalence of delusional (Abu‐Akel, 1999; Frith & Corcoran, 2009), disorganized (Hardy‐Baylé *et al*., 2003), hallucinatory (Frith & Corcoran, 2009), and negative symptoms (Shamay‐Tsoory, Shur, Barcai‐Goodman, Medlovich, Harari & Levkovitz, 2007).

We consider the improvement in higher‐order and overall ToM important, as these are the most complex operations, which require the most comprehensive and recursive thinking and perspective taking (Choudhury, Blakemore & Charman, 2006; Valle, Massaro, Castelli & Marchetti, 2015). Complex mind‐reading abilities evolve after childhood; thus, appropriate adjustment to peers and adequate communication with them require complex social skills (Blakemore & Choudhury, 2006; Brizio, Gabbatore, Tirassa & Bosco, 2015). This is true not only in adolescence, but also later in life. Consequently, improvements in this area can be of paramount importance for the proper social adaptation of schizophrenic patients. Moreover, it can contribute to symptom severity reduction.

### Subjective acceptance and feasibility

MCT participants reported good subjective applicability and acceptability. The fact that participants appreciated the training, and considered it to be clearly useful, can be regarded momentous, knowing the motivational difficulties and lower level of adherence and cooperation among patients diagnosed with schizophrenia. All these results are further supported by the drop‐out rate, as no intervention group member left the study prematurely, compared to the group only receiving TAU.

The patients in our trial showed excellent adherence, as reflected by the fact that all the members of the intervention group completed the 16‐week training; all of them agreed to participate in the 6‐month follow‐up assessments, and attended 97.55% of the group settings.

The only adverse event that occurred during the study is worth examining from several perspectives. On the one hand, the development of depressive symptoms as a side effect was obviously an unpleasant event; on the other hand, it might have been trigerred by the patient's increased awareness, which can be interpreted as a sign of metacognitive awareness of impaired social cognitive functions. It is noteworthy that after recovery the patient was able to utilize this awareness as motivation, and the event did not lead to hopelessness.

In addition to our results, the trial has many limitations. First, after losing one of the sites, the sample size became relatively small. Second, in order to eliminate learning effect, it would have been worthwhile to reassess neurocognitive functions only at T2. Third, the follow‐up period of 6 months is relatively short, and should be extended to further investigate the persistence of the effects we found. Finally, a study design with fewer output variables could reduce the possibility of type I error.

## CONCLUSION

Our results tend to be in line with previous studies on the improvement of symptom severity in schizophrenia in patients who received MCT. We found that participants who had more severe symptoms at the start of the training benefited more from MCT in terms of overall symptom severity. In addition, the training method led to improvements in subdomains of neurocognitive and social cognitive functioning. However, it should be noted that we found positive changes in only a few neurocognitive and social subdomains, and changes in social cognitive functioning were detectable only within the intervention group, which calls for a cautious interpretation of our results; as does the fact, that there was a notable reduction in the number of significant findings after correction on multiple comparisons.

Patients showed an excellent adherence to the training, and their subjective perception of it was positive; they found it particularly useful and interesting.

Only one adverse event occurred (in the form of depressive symptoms), which, however, was rooted in the patient's increased metacognitive awareness.

The authors express their gratitude to Professor Steffen Moritz for all his help, and Emőke Takácsné Tóth for improving the use of English in the manuscript.

The Hungarian National Research Ethics Committee approved the study protocol in line with the Helsinki Declaration. All patients signed an informed consent form, and were assured of anonymity and data confidentiality. Registration number: 13175‐2/2017/EKU.

## Supporting information


**Appendix S1**. Supplementary Information.
**Fig. S1** Flow diagram of the stratified randomization process. PANSS: Positive and Negative Syndromes Scale, MCT: Metacognitive Training, TAU: treatment as usual.
**Table S1** Results on Akaike’s information criterion separately for within‐group and between‐group tests, and for T0‐T1 and T1‐T2. Bold: best model fit, i.a.: interaction. PANSS: Positive and Negative Symptoms Scale, WCST: Wisconsin Card Sorting Test, RBANS: Repeatable Battery for the Assessment of Neuropsychological Status, RMET: Reading the Mind in the Eyes Test, ToM PST: Theory of Mind Picture Stories Task

## Data Availability

Our data are available in (Fekete, Vass, Balajthy *et al*., 2021b).
