# Supplementary material for: Efficacy of metacognitive training on symptom severity, neurocognition and social cognition in patients with schizophrenia: A single‐blind randomized controlled trial
Source: Scand J Psychol. 2022 Apr 6;63(4):321–33. doi: 10.1111/sjop.12811 (PMC9544200; doi:10.1111/sjop.12811)
Supplement: Supplementary file 1 — Appendix S1. Supplementary Information. Fig. S1 Flow diagram of the stratified randomization process. PANSS: Positive and Negative Syndromes Scale, MCT: Metacognitive Training, TAU: treatment as usual. Table S1 Results on Akaike’s information criterion separately for within‐group and between‐group tests, and for T0‐T1 and T1‐T2. Bold: best model fit, i.a.: interaction. PANSS: Positive and Negative Symptoms Scale, WCST: Wisconsin Card Sorting Test, RBANS: Repeatable Battery for the Assessment of Neuropsychological Status, RMET: Reading the Mind in the Eyes Test, ToM PST: Theory of Mind Picture Stories Task [file SJOP-63-321-s001.docx]

SUPPLEMENTARY MATERIAL

of the paper entitled

**Efficacy of Metacognitive Training on Symptom Severity, Neurocognition and Social Cognition in Patients with Schizophrenia: a Single-Blind Randomized Controlled Trial**

Supplement A

The following section provides a detailed description of the process of stratified randomization (Figure 1).

1. Symptom severity was defined on the basis of PANSS total scores. For this purpose the recommendations of Leucht et al. (2005) were used, who found that participants can be divided into four groups regarding symptom severity along cut-off scores. In our present study, we only focused on the 75 point cut-off score of Leucht et al., which divides patients into two groups based on symptom severity. According to Leucht et al., patients with PANSS total scores below 75 fell into the mildly or moderately ill category, while patients with scores above 75 were classified as markedly or severely ill. We created two groups along the 75 PANSS total value. Finally, thirty-one of the participants showed PANSS total scores ≥ 75, and eighteen of them had scores <75.
2. Using their identification number, patients from the two groups formed on the basis of symptom severity were randomly assigned into the intervention group or the control group with the help of the online software Research Randomiser (Urbaniak & Plous, 2013), separately at each study site.
3. Patients randomized to participation in the training (from both the PANSS total ≥ 75 and the PANSS total < 75 group) were merged into one intervention group at the site, and the same was done for the control group. This way study groups with equal symptom severity ratios were formed. Overall, three patients quit the study during the randomization process; however, the frequency of symptom severity remained statistically equal across the TAU and TAU+MCT groups (ꭓ^2^(1, 46)= 0.383, p= 0.536) with twenty-three patients in both groups.

Patients assessed with the help of the PANSS scale (n=49)

Allocated to control group

(TAU)

(n= 23)

Allocated to intervention group (TAU+MCT)

(n= 23)

Declined to participate

(n= 2)

Allocated to control group (TAU)

(n= 7)

Declined to participate

(n= 1)

Allocated to intervention group (TAU+MCT)

(n= 14)

Randomized to intervention group (TAU+MCT)

(n= 15)

Randomized to control group (TAU)

(n= 16)

Randomized to intervention group (TAU+MCT)

(n= 9)

Randomized to control group (TAU)

(n= 9)

≥ 75 PANSS total score

(n= 31)

< 75 PANSS total score

(n= 18)

**Figure 1.** Flow diagram of the stratified randomization process

PANSS: Positive and Negative Syndromes Scale, MCT: Metacognitive Training, TAU: treatment as usual

Supplement B

The table contains the detailed results on Akaike’s information criterion (AIC). Lower AIC values indicate a better model fit.

|  | **T0-T1 Within groups** | | **T1-T2 Within groups** | | **T0-T1 Between groups** | | | | **T1-T2 Between groups** | | | |
| --- | --- | --- | --- | --- | --- | --- | --- | --- | --- | --- | --- | --- |
|  |  |  |  |  | **random intercept** | | **fixed intercept** | | **random intercept** | | **fixed intercept** | |
|  | **fixed intercept** | **random intercept** | **fixed intercept** | **random intercept** | **without i.a. terms** | **with**  **i.a. terms** | **without i.a. terms** | **with**  **i.a. terms** | **without i.a. terms** | **with**  **i.a. terms** | **without i.a. terms** | **with**  **i.a. terms** |
| ***Symptom severity***  **PANSS** | | | | | | | | | | | | |
| Positive | **310.355** | 312.908 | **290.928** | 295.907 | 540,235 | 535,993 | 535.718 | **533.699** | 450.888 | 447.765 | 448.624 | **445.497** |
| Negative | **323.234** | 325.788 | **304.008** | 308.987 | 563,022 | 557,638 | 556.151 | **552.984** | 472.685 | 469.117 | 470.420 | **466.848** |
| Disorganized | **311.490** | 314.043 | **300.315** | 305.294 | 560,610 | 551,375 | 553.739 | **546.721** | 478.536 | 474.774 | 476.272 | **472.505** |
| Excitement | **291.462** | 294.016 | **263.144** | 268.122 | 503,763 | 500,200 | 496.892 | **495.546** | 412.230 | 409.558 | 409.966 | **407.290** |
| Emotional distress | **310.925** | 313.478 | **289.504** | 294.483 | 539,564 | 535,683 | 535.693 | **531.029** | 451.842 | 448.612 | 449.577 | **446.343** |
| Total | **407.022** | 409.575 | **378.383** | 383.362 | 700,981 | 694,973 | 698.754 | **692.679** | 591.196 | 585.981 | 588.932 | **583.712** |
| ***Neurocognition***  **Executive functions (WCST-64)** | | | | | | | | | | | | |
| Total error | **343.815** | 346.401 | **343.812** | 348.791 | 617,970 | 612,477 | 611.043 | **607.803** | 530.228 | 523.322 | 527.963 | **521.054** |
| Perseverative error | **287.989** | 290.575 | **291.787** | 296.766 | 235,312 | **235,312** | 503.391 | 500.682 | 424.944 | 421.957 | 422.680 | **419.688** |
| **Further neurocognitive functions (RBANS)** | | | | | | | | | | | | |
| Immediate memory | **302.592** | 315.146 | **304.864** | 307.290 | 551,524 | 547,885 | 544.653 | **543.231** | 478.813 | 475.550 | 476.553 | **473.286** |
| Visuospatial functions | **259.423** | 261.976 | **220.765** | 223.191 | 469,698 | 466,618 | 462.827 | **460.964** | 371.326 | 369.330 | 369.066 | **367.066** |
| Language | **288.156** | 290.709 | **281.060** | 283.485 | 507,116 | 503,523 | 500.245 | **498.869** | 440.226 | 437.115 | 437.967 | **434.851** |
| Attention | **335.496** | 338.049 | **331.815** | 334.241 | 630,389 | 620,987 | 623.517 | **616.333** | 529.326 | 524.524 | 527.066 | **522.260** |
| Delayed memory | **294.338** | 296.891 | **297.856** | 300.281 | **522,104** | 529,740 | 525.233 | 524.086 | 461.189 | 457.734 | 458.929 | **455.470** |
| ***Social cognition***  **Emotion recognition (RMET)** | | | | | | | | | | | | |
| Emotion recognition | **268.248** | 270.801 | **260.323** | 262.749 | 458.732 | 456.148 | 451.840 | **451.484** | 402.549 | 397.477 | 400.281 | **395.204** |
| **Theory of mind (ToM PST)** | | | | | | | | | | | | |
| Sequencing | **296.178** | 298.732 | **281.787** | 284.212 | 519.844 | **416.755** | 513.000 | 512.091 | 441.827 | 438.733 | 439.563 | **436.465** |
| Theory of a single person’s mind | **220.898** | 223.452 | **202.389** | 204.815 | 380.756 | 379.453 | **373.872** | 374.789 | **304.740** | 313.171 | 312.476 | 310.902 |
| Switching between minds | **146.571** | 149.124 | **131.743** | 134.169 | 277.767 | 274.154 | 270.883 | **269.491** | 220.347 | 215.302 | 218.083 | **213.033** |
| Comprehension of misleading | **159.114** | 161.668 | **123.697** | 148.608 | 298.970 | 297.310 | 292.886 | **292.646** | 232.842 | **231.753** | 230.578 | **229.485** |
| Total | **334.969** | 337.523 | **319.878** | 322.304 | 578.928 | 574.814 | 572.044 | **570.150** | 494.506 | 490.829 | 492.242 | **488.560** |

**Table 1.** Results on Akaike’s information criterion separately for within-group and between-group tests, and for T0-T1 and T1-T2

**bold:** best model fit, i.a.: interaction

PANSS: Positive and Negative Symptoms Scale, WCST: Wisconsin Card Sorting Test, RBANS: Repeatable Battery for the Assessment of Neuropsychological Status, RMET: Reading the Mind in the Eyes Test, ToM PST: Theory of Mind Picture Stories Task
